# Editorial Notes

**Published:** 1949-10

**Authors:** 


### Editorial Notes

**Domiciliary Consultations.**

The pattern of the National Health Service is gradually taking shape, at least sufficiently to show what modifications will first require attention. It is now even rumoured that Consultants will be offered their "contracts" at an early date! We hear, however, that very little use is being made of that part of the service which provides a consultation at the patient's home if he is unable to attend hospital. This arrangement is obviously a great advantage in many cases. We may remind our readers that it is available to *anyone*, even though he is not on the "list" of any general practitioner.

**Unusual Children.**

A large part of this issue is devoted to "Unusual Children", a collection of cases illustrating a number of conditions seldom encountered in practice and therefore presenting special difficulty in diagnosis. Our purpose is to help the family doctor to reach a diagnosis, or at least a probable diagnosis, without undue delay, and in the main by clinical means. Obviously in many cases further investigation and perhaps consultation with an experienced pædiatrician will be necessary before the diagnosis can be considered complete. But since delay is often a matter of great importance to the patient, we hope that by stimulating interest in a difficult but fascinating branch of medicine we shall serve his interests as well as our readers'. Much of the value of such a collection consists in the contrast of one type against another. Compare, for example, the two jolly little fellows with Brailsford dwarfism, swollen joints but normal intelligence, and the three miserable, depressed and mentally retarded children suffering from gargoylism.

Progeria is a rare and weird condition, but it appears (of all places) in the *Bab Ballads*! There it is associated with sexual precocity, not observed in the other cases reported:

> "He early determined to marry and wive . . .
> Which the poor little boy didn't live to contrive
> His health didn't thrive—
> No longer alive
> He died an enfeebled old dotard at five!"

It is notable that mongols and the sufferers from gargoylism usually also die in childhood. On the other hand, congenital syphilis and cretinism respond remarkably to appropriate treatment, which emphasizes the importance of early diagnosis.

### Obituaries

